# A cultured approach to canine urothelial carcinoma: molecular characterization of five cell lines

**DOI:** 10.1186/s40575-015-0028-3

**Published:** 2015-09-17

**Authors:** SG Shapiro, DW Knapp, Matthew Breen

**Affiliations:** Department of Molecular Biomedical Sciences, College of Veterinary Medicine, North Carolina State University, 1060 William Moore Drive, Raleigh, NC 27607 USA; Department of Veterinary Clinical Sciences, Purdue University, College of Veterinary Medicine, West Lafayette, IN USA; Purdue University Center for Cancer Research, West Lafayette, IN USA; Center for Comparative Medicine and Translational Research, North Carolina State University, Raleigh, NC USA; Center for Human Health and the Environment, North Carolina State University, Raleigh, NC USA; Lineberger Comprehensive Cancer Center, University of North Carolina, Chapel Hill, NC USA

**Keywords:** Transitional cell carcinoma, Bladder cancer, Urothelial carcinoma, Cell lines, Lipid metabolism, Chromosome

## Abstract

**Background:**

Urothelial carcinoma (UC), also known as transitional cell carcinoma (TCC), of the bladder is the most common neoplasm affecting the canine urogenital system. To facilitate study of the disease *in vitro,* cell line models have been established from primary tumor biopsies. Their resemblance to the primary disease, however, has not been well defined. In the present study, we evaluated five canine UC cell lines via oligonucleotide array comparative genomic hybridization (oaCGH), fluorescence *in situ* hybridization (FISH), and gene expression analysis.

**Results:**

Comparison of genome wide DNA copy number profiles of the cell lines with primary biopsy specimens revealed redundancies in genomic aberrations, indicating that the cell lines retain the gross genomic architecture of primary tumors. As in the primary tumors, gain of canine chromosomes 13 and 36 and loss of chromosome 19 were among the most frequent aberrations evident in the cell lines. FISH analysis revealed chromosome structural aberrations, including tandem duplications, bi-armed chromosomes, and chromosome fusions, suggesting genome instability during neoplastic transformation. Gene expression profiling highlighted numerous differentially expressed genes, including many previously shown as dysregulated in primary canine UC and human bladder cancer. Pathway enrichment analysis emphasized pathways suspected to be at the crux of UC pathogenesis, including xenobiotic and lipid compound metabolism.

**Conclusions:**

These data support valid use of the canine UC cell lines evaluated by confirming they provide an accurate and practical means to interrogate the UC at a molecular level. Moreover, the cell lines may provide a valuable model for furthering our understanding of aberrant metabolic pathways in UC development.

**Electronic supplementary material:**

The online version of this article (doi:10.1186/s40575-015-0028-3) contains supplementary material, which is available to authorized users.

## Background

Urothelial carcinoma (UC), also referred to as transitional cell carcinoma, is the most common bladder neoplasm in the dog [1]. While precise lifetime risk and incidence numbers in pet dogs are unknown, UC is estimated to affect more than 20,000 dogs each year in the United States [2]. Due to the uncomfortable and potentially fatal consequences associated with a rapidly growing bladder tumor, the diagnosis of UC conveys a guarded prognosis and evokes considerable dog owner concern [3]. Additionally, over 90 % of canine bladder tumors are invasive with metastatic potential; approximately 20 % of canine patients show overt metastases at the time of diagnosis and over 60 % at death [2]. The devastating clinical course of disease, combined with a high rate of metastasis, emphasizes a need for early detection of the tumor. With improved detection and a better understanding of the disease, therapeutics may prove more efficacious, extend patient survival, and improve quality of life.

Previous studies have shown that the genomic landscape of canine UC is highly aberrant, with recurrent chromosome copy number aberrations affecting gene dosage on, most notably, canine chromosomes (CFA) 13, 19, and 36 [4]. In addition, overwhelming breed predispositions suggest the risk for UC development is at least partially genetic. The inbred history of purebred dogs, resulting in reduced levels of genetic variation and enrichment in breed-associated loci, provides a biologic basis for breed susceptibility [5]. Scottish terriers, for example, are diagnosed with UC 20 times more frequently than the average of all breeds, and Beagles, Shetland sheepdogs, Wire hair fox terriers, and West Highland white terriers are reported to be affected three to five times as frequently [1]. The uniquely homogenous genetic population of purebred dogs makes the dog an excellent model for teasing out genetics that are potentially important in cancers of the highly heterogeneous human population. Previous studies also have found that canine UC closely resembles that of the human counterpart, in histopathological features, clinical behavior, and genomic aberrations, furthering the value of the dog to human medicine [2, 6, 4].

In addition to genetic risk factors, UC is associated with numerous environmental factors, including flea and tick dips, obesity, pesticides, and cyclophosphamide treatment [7–9]. While correlations of UC incidence with each of these risk factors have been found, mechanisms of carcinogenesis have yet to be elucidated. Problems in cyclic compound metabolism have been proposed, but until the mechanisms of UC development are elucidated, the provision of effective prevention and treatment strategies will remain challenging [10]. The study of potential molecular drivers and therapeutic targets would be greatly expedited by a biologically appropriate *in vitro* model.

Cell lines provide a useful *in vitro* model for the study of disease. By facilitating characterization and manipulation of all molecular facets without harming the model animal, cell lines enable researchers to evaluate highly experimental ideas and therapies. Although canine UC cell lines have previously been established for such use [11], their resemblance to the primary tumor in the context of molecular behavior and genomic landscape has yet to be thoroughly evaluated. Affirming the cell lines recapitulate molecular characteristics of the primary tumor would increase their clinical predictive value and enhance their significance in translational studies.

In the present study, we investigated the molecular profile of five canine UC cell lines with oligonucleotide array comparative genomic hybridization (oaCGH) and fluorescence *in situ* hybridization (FISH). Clinical relevance of findings was validated by comparison of oaCGH profiles of cell lines with those of primary tumor samples, which showed conservation of major genomic aberrations in both sample cohorts. Subsequent gene expression profiling quantified mRNA from copy number neutral and aberrant regions of the genome. KEGG pathway and gene ontology (GO) analysis demonstrated that genes involved in lipid metabolism and cell cycle regulation were enriched among genes differentially expressed between neoplastic cell lines and healthy urothelium, highlighting a potential pathogenesis that is relevant to known risk factors. Our results affirm that the canine UC cell lines are genomically similar to the primary tumor, providing a relevant *in vitro* model for study of the molecular mechanisms of disease.

## Methods

### Case collection

Five tumor cell lines (Table 1) were established from pathologically-confirmed UC in dogs who were evaluated and treated at the Purdue University Veterinary Teaching Hospital. The biopsies used to establish the cell lines were collected via cystoscopy or at necropsy. All samples were obtained with informed pet owner consent and under an approved IACUC protocol. Once established, cells from each line were cryopreserved at various passages.

**Table 1:** Characteristics of five canine UC cell lines

Cell Line ID	Initial passage	Breed	Sex	Average chromosome #	Specific structural changes
K9TCC-PU-An (TCC2)	p3	Scottish Terrier	FS	131.6	TD(10,36), t(36;38),f(13;13)
K9TCC-PU-In (TCC6)	p1	German Shepherd	FS	103.3	TD(10,36), f(13;13)
K9TCC-PU-Mx (TCC5)	p5	German Shepherd	FS	134.6	TD(10,36)
K9TCC-PU-Sh (TCC4)	p6	Collie	FS	105.5	TD(36,38), t(36;38),
K9TCC (TCC3)	p14	Mixed Breed	FS	76.2	TD(10)

Five cell lines, derived from primary biopsy specimens, were evaluated in this study. Cell line identifier, initial passage number, breed, and sex (*FS* female spayed) are shown. Chromosomes from metaphase spreads (*n* = 30) of each cell line were counted to determine an average chromosome number. Recurrent structural changes identified by FISH evaluation are shown (*TD* tandem duplication, *t* translocation, *f* fusion)

Control samples for expression analysis (healthy urothelium) were collected during necropsy of dogs with no clinical or histopathological signs of disease at North Carolina State University College of Veterinary Medicine. After collection, bladder tissue was stored in transport media (RPMI media supplemented with 10 % fetal bovine serum (FBS) until sample processing (<1 h). Samples were split in half: one half was fixed in 10 % neutral buffered formalin (NBF) before paraffin-embedding (FFPE) to enable pathologic evaluation, and the second half was used for urothelial cell isolation. FFPE samples were sectioned and stained with hematoxylin and eosin (H&E) for review by a board certified veterinary pathologist. All control samples were found to be histopathologically normal prior to use in subsequent protocols. Urothelial cells were isolated by scraping the bladder mucosa three times with a glass slide and rinsing with 1x phosphate buffered saline (PBS), as described previously [12]. Urothelial scraping was performed in order to minimize contamination of urothelium by submucosal tissue layers. Cell scrapings were then analyzed under phase contrast to determine the proportion of epithelial cells to contaminating mesenchymal cells. Over 90 % of isolated cells were confirmed to be epithelial.

### Culture preparation

Cells from each of the five lines were cultured in DMEM/F12 without glutamine (Mediatech, Manassas, VA) and supplemented with 10 % FBS (Mediatech), 1 % Glutamax™ (Life Technologies, Carlsbad, CA), and 0.6 % Primocin™ (InvivoGen, San Diego, CA) to confluence, passaging when confluent. For each line, cells from the same flask were split into three aliquots and used simultaneously for chromosome preparation, DNA isolation, and RNA isolation. Four of the cell lines (K9TCC-PU-An, K9TCC-PU-In, K9TCC-PU-Mx, K9TCC-PU-Sh) were available as low passage (<p7) and so were harvested at an earlier (<p10) and later (>p16) passage to evaluate genomic evolution during culture. One cell line (K9TCC) was available only from p14 and so was harvested at p16 only.

### oaCGH

DNA from each cell line ("test") was isolated using a DNeasy Kit according to manufacturer’s recommendations (Qiagen, Valencia, CA). Purified DNA was verified to be of high molecular weight and purity by agarose gel electrophoresis and spectrophotometry (Nanodrop™ 1000, Thermo Fisher Scientific, Wilmington, DE; 260:230 > 2.0 and 260:280 > 1.8). Since all five cell lines were female, a single female "reference" mix was generated by combining equimolar quantities of DNA extracted from ten different, healthy female dogs of various breeds (QIAmp DNA Midi Kit, Qiagen). Isolated DNA was labeled using the Genomic DNA Enzymatic Labeling Kit (Agilent Technologies, Santa Clara, CA) as described previously [4, 13]. Fluorescently labeled test and reference DNA samples were hybridized to Canine G3 Sureprint 180,000 feature oaCGH arrays (Agilent, AMADID 025522) for 40 h, as described previously [13, 14]. Arrays were scanned at 3 μm (Agilent, Model G2505C) and data extracted with Feature Extraction software (v10.9  Agilent) using the canFam2 genome build. CGH results were analyzed using the FASST2 algorithm in Nexus Copy Number (Biodiscovery, Hawthorne, CA). After aberration detection, cell lines and primary tumors were clustered based on aberrations using a complete linkage hierarchical clustering algorithm by Nexus Copy Number (Biodiscovery).

Genomic regions likely to be central to tumorigenesis were pinpointed by the Genomic Identification of Significant Targets in Cancer (GISTIC) algorithm in Nexus Copy Number, which identifies regions unlikely to be aberrant by chance when taking aberration frequency and amplitude into account [15]. A G-score is computed to reflect the frequency and amplitude of the aberration, while a q-value suggests the likelihood (probability) of that aberration occurring by chance when looking at the overall aberration pattern throughout the genome. Lastly, aberrations in early and late passage cell lines were compared using the “Comparisons” tool in Nexus, using the early passage as baseline (*p* = 0.05, differential threshold = 1 %).

### Primary tumor oaCGH data

Previously published oaCGH data from 31 primary canine UCs was compared to cell line aberrations [4]. None of the cell lines used in the current study were derived from any of the 31 primary tumors. Using Nexus Copy Number, aberrations common to primary tumors and cell lines were compared using the “Comparisons” tool. Additionally, GISTIC analysis was repeated using primary tumor aberrations.

### Fluorescence *in situ* hybridization (FISH)

Cells were harvested using conventional methods of colcemid-induced metaphase arrest (Life Technologies), hypotonic (KCl) treatment (Life Technologies), trypsinization (0.25 % trypsin, Mediatech), and fixation, as described previously [16]. Fixed cell suspensions were dropped onto clean glass slides and aged for three days at room temperature prior to ethanol dehydration (70 %/90 %/100 %) and storage at −80 °C.

Fluorescence *in situ* hybridization (FISH) was used to validate and visualize copy number aberrations indicated by oaCGH. Based on FASST2-called aberrations, 11 genomic regions were selected for FISH analysis: 10 aberrant regions of high frequency (gained/lost in at least three cell lines) and one balanced region on CFA 11, selected as a copy number neutral control (Log_2_ = 0) (Table 2). Probe DNA was extracted from clones of the CHORI-82 canine BAC library (http://bacpac.chori.org/library.php?id=253) containing the regions of interest and labeled with one of five spectrally-resolvable fluorochrome-conjugated dNTPs as described previously [17]. Multicolor FISH reactions were performed first on DAPI-stained metaphase chromosome preparations of clinically healthy dogs to validate the unique and precise cytogenetic location of each probe, as described previously [16]. Verified probes were hybridized to metaphase preparations of each cell line and visualized by using an Olympus BX61 fluorescent microscope (Olympus, Center Valley, PA) equipped with appropriate single pass filters. Cells (n ≥ 30) exhibiting good chromosome separation were selected for assessment of numerical and structural organization and were counted to determine a) total chromosome number and b) number of signals for each fluorochrome (sequence of interest).

**Table 2:** Regions chosen for FISH validation of oaCGH

BAC address	Region probed	Fluorochrome	oaCGH-called aberration
326 N03	chr1:35846825-36021610	Red	Loss
307I06	chr6:49193262-49385361	Green	Loss
326H08	chr10:13936488-14128322	Cy5	Amplification
126 F01	chr12:52174704-52401789	Gold	Loss
186 J06	chr13:38344516-38552798	Aqua	Gain
332 N02	chr19:31345273-31534341	Red	Loss
313D22	chr21:5212350-5387435	Green	Loss
307D14	ch33:5409680-5589439	Red	Loss
199 F16	chr36:7057670-7276062	Cy5	Amplification
328D10	chr38:25164963-25365710	Gold	Amplification
326 L05	chr11:20496757-20706739	Aqua	Neutral

Ten BAC clones were selected to probe regions shown to be highly aberrant in the cell lines (>50 % penetrant). A copy number neutral region on CFA11 was chosen as an internal control to ensure correct interpretation of relative locus copy number. The BAC address, genomic location (CanFam2.0 assembly), and associated fluorochrome for each probe are shown

Using Log_2_ ratios ascertained by oaCGH and also deduced from single locus-BAC probe probe enumeration, correlation coefficients were derived. To calculate the Log_2_ ratio, the average copy number of the aberrant locus was divided by the average copy number of the CFA11 neutral locus of each cell line. A Log2 of the resultant value was calculated and used as the FISH-derived Log2 for each copy number-aberrant locus. Additionally, a Mann–Whitney *U* test (two-tailed, *p* < 0.05) was performed using JMP Professional Statistical Software (v. 11, SAS, Cary, NC) to compare mean Log_2_ ratios from both methods.

### Gene Expression

RNA was extracted from cultured cells or control urothelial cells using the RNeasy Plus Mini Kit (Qiagen). RNA was immediately isolated from the healthy, fresh urothelial cells to minimize alterations in post-mortem and post-collection gene expression. RNA purity and integrity was assessed using spectrophotometry (Nanodrop) and a 2100 BioAnalyzer (Agilent), respectively. Samples with 260:230 and 260:280 > 2.0 and RIN > 8.0 were used for microarray analysis and qRT-PCR validation. RNA was labeled using the Quick Amp Labeling kit (Agilent). Purified RNA probes were hybridized to a one-color expression microarray (Agilent SurePrint G3 Canine 4x44k Expression Array). Arrays were scanned at 3 μm (Agilent, Model G2505C). Analysis was performed using the GeneSpring advanced platform (Agilent, v.11.5, 2011) and Nexus Expression (BioDiscovery, v.2.0, 2010). Prior to expression normalization, compromised probes were removed from analysis in GeneSpring, including features which were non-uniform, saturated, or population outliers. Normalized signal values were obtained by log transformation, followed by 75th percentile shift normalization and baseline shift to median of all samples. Normalized signal values (NS) were compared between RNA isolated from the cell lines and from urothelium of two histologically confirmed healthy dog bladders to determine the fold change in expression (NS_cell_line_ ÷ NS_control_average_ = relative fold change_gene_). Significantly over- and underexpressed genes in demonstrated a ± 2-fold change in gene expression in the cell lines relative to the average of control bladders. The resulting gene expression profiles of cell lines and healthy control urothelium were clustered using an agglomerative, unsupervised hierarchical clustering algorithm in Nexus Expression (Biodiscovery).

In preparation for qRT-PCR validation of array data, primers were designed for a stably expressed control gene (*RPL32*) and for a gene within each of the loci assessed by FISH and dysregulated according to the expression array. Ct values for *RPL32* were within 0.7 Ct among all cell lines and controls (standard deviation = 0.23), validating its use as a reference gene. Ct Primer template specificity was confirmed by agarose gel electrophoresis of PCR products (single product) and primer melt curve analysis (single peak). An efficiency curve based on five ten-fold dilutions was constructed to evaluate the performance of each primer pair. Primers with efficiency between 90-110 % and linear correlation coefficients over 0.95 were selected for qRT-PCR analysis (seven aberrant regions of interest plus a control gene, Table 3). cDNA was created using the QuantiTect Reverse Transcription Kit (Qiagen). The Quantifast SYBR Green qPCR Kit (Promega, Madison, WI) was used for real time quantification of mRNA on an iCycler iQ™ Real Time Detection System (Bio-Rad, Hercules, CA). Fidelity was ensured by inclusion of no reverse transcriptase and no template negative controls. A correlation coefficient relating data from qRT-PCR and expression array was calculated using fold change values from genes evaluated using both methods. Gene ontology (GO) and pathway analysis was conducted on differentially expressed genes (DEGs) using the online tool DAVID to extract biological relevance from the data [18].

**Table 3:** Copy number aberrant genes analyzed by qRT-PCR

Gene	Location	Forward primer	Reverse primer
*RNPC3*	chr6:50072550-50098045	*5′-GGGGCGACCGGCCCTTCTA-3′*	*5′-GACAGGACGCGCACCGACTG-3′*
*MDM2*	chr10:13920606-13946580	*5′-ACGGCAGAGAAAGCGCCACAAA-3′*	*5′-GGCGTCCCTGTTGACTCACTG-3′*
*EIF2C2*	chr13:38222456-38271623	*5′-CAAAGGCAGTCCAGGTTCAT-3′*	*5′-GGGCATCTGTTGGTCTGAGT-3′*
*RALB*	chr19:32842484-32862087	*5′-GTGTTCTTGCTCTCCCCAAC-3′*	*5′-TCCAAAACCTCCCAACAAAG-3′*
*CLNS1A*	chr21:24140052-24163815	*5′-CCTGTGTCTCCGCGCTCCCTG-3′*	*5′-GCCTCGGTCTCGGGCTGCTG-3′*
*STX19*	chr33:4727847-4728760	*5′-CTGCTATGTTCCGCCAATTT-3′*	*5′-GCACTTCTTTTCCAGCAACC-3′*
*TAGLN2*	chr38:25240217-25241796	*5′-TGCGGACCTGGAGCAGATCCTG-3′*	*5′-ACACAGCACCGTGCCATCCT-3′*
*RPL32*	chr18:10198892-10204028	*5′-ATGCCCAACATTGGTTATGG-3′*	*5′-ATGCCCAACATTGGTTATGG-3′*

The mRNA levels of genes located within seven regions of copy number aberration were evaluated by qRT-PCR. The targeted gene, genomic location (CanFam2.0 assembly), and primer pair utilized are shown

## Results

### Canine UC cell lines display recurrent chromosome copy number and structural aberrations

**Fig. 1:**
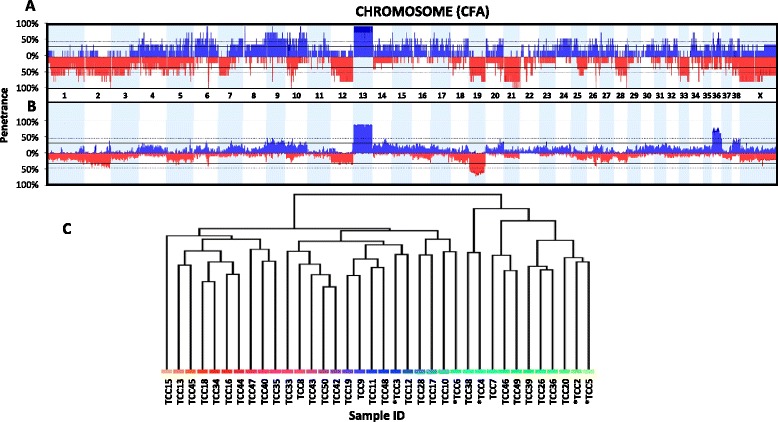
oaCGH profiles of five canine UC cell lines recapitulates those seen in 31 primary tumors. Cell lines were analyzed via oaCGH at an early stage (<p16) and aberrations called based on Log_2_ ratios relative to normal canine DNA (**a**). When compared with 31 primary tumors previously analyzed (**b**), aberrations were shared between the two cohorts, validating the use of canine UC cell lines in genomic studies. When clustered by oaCGH aberration profiles (**c**), the cell lines (astericked; TCC2 = K9TCC-Pu-An, TCC3 = K9TCC, TCC4 = K9TCC-Pu-Sh, TCC5 = K9TCC-Pu-Mx, TCC6 = K9TCC-Pu-In) clustered among the primary tumors (no asterisk) rather than appearing as an outgroup, further showing their greater similarity to the primary tumors than to one another and showing minimal effect of the culture environment on genome evolution

Metaphase preparations from each of the five canine UC cell lines revealed a high degree of aneuploidy, with an excess of 100 chromosomes in four of five cell lines (Table 1). Additionally, oaCGH profiles revealed specific DNA copy number gains and losses across the genome of each cell line (Fig. 1a). Highly recurrent aberrations (>80 % frequency) involved loss of regions of CFA 1, 2, 5, 6, 9, 10, 12, 19, 20, 21, 22, 26, 28, 33, 34, and X, and gains of CFA 4, 5, 6, 8, 9, 10, 11, 13, 15, 16, 17, 27, 34, and 36. Shared copy number losses present on CFA 2, 6, 10, 21, 26, and 28 and gains of CFA 6, 8, 10, and 13 were observed in all five cell lines (100 %). High amplitude aberrations (Log_2_ > 4) were noted on CFA10, 36, and 38, suggesting DNA amplification in these regions. To identify regions differentially aberrant between early and later passage cell lines, a “comparisons” analysis was performed in Nexus Copy Number. The analysis showed only three genomic regions where the aberration frequency among the early and later passage cell lined differed by more than 1 %: CFA1q22, CFA2q24.1, and CFA9q22.3 (*p* < 0.01). Although copy number neutral in early cell line passages, later passages of the same cell lines demonstrated a copy number gain.

Cell line oaCGH profiles were compared to those of 31 previously published primary tumors (Fig. 1b). Hierarchical clustering of chromosome copy number aberrations among cell lines and primary tumors yielded a dendogram in which there was no segregation between the cell lines and primary tumors (Fig. 1c). Comparison analysis of primary tumors and cell lines highlighted only 17 regions, constituting less than 0.2 % of the genome (4.5 Mb), aberrant in either the primary tumors or cell lines, while normal in the other (Additional file 1: Table S1). GISTIC analysis of aberrations among primary tumors and cell lines showed that 71.4 % (five of seven total GISTIC-identified regions) of specific cancer-associated genomic regions aberrant in the cell lines were similarly altered in the primary tumors, including amplified regions on CFA10, 36, and 38 and gains of CFA13 (Q-bound < 0.005, G-score > 5, Table 4). The primary tumors were generally more aberrant, with 60 significant cancer-associated genomic regions identified. Thus, GISTIC aberrations among cell lines represent only 8.3 % of those in the primary tumor.

**Table 4:** Regions implicated by GISTIC analysis represent regions relevant to neoplasia

Genomic region	Aberration	Q-Bound	G-Score
chr10:13,614,516-14,400,849	CN Gain	8.22E-06	9.606238
chr10:17,743,915-20,676,296	CN Loss	0.02737	7.847499
chr10:44,389,882-71,751,790	CN Gain	0.005379	5.952698
chr10:5,318,483-7,102,150	CN Gain	0.005379	5.990278
chr13:3,033,844-66,047,830	CN Gain	0.005379	5.576475
chr36:22,492,857-25,421,734	CN Gain	0.005379	5.578811
chr38:3,874,100-4,368,891	CN Gain	0.005379	5.403421

Minimum regions of shared aberration among cell lines are shown, as well as the called aberration (*CN* copy number), Q-bound, and G-score. The G-score reflects the frequency as well as magnitude of the aberration in the cohort, while the Q-bound indicates significance. The most notable regions were located on CFA10, CFA13 and 36; gains of CFA13 and 36 are of diagnostic relevance in primary UC and were similarly aberrant in cell lines

**Fig. 2:**
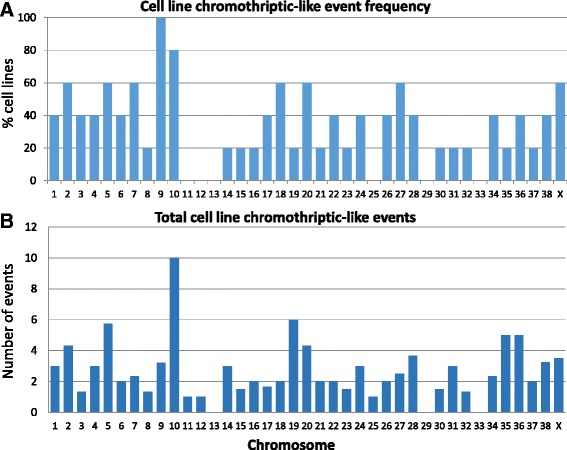
The cell lines demonstrate evidence of chromothriptic-like events in tumor evolution. Chromothriptic-like events were seen throughout the genomes of all five cell lines, involving all but six chromosomes (**a**). The maximum average number of events on a single chromosome was observed on CFA10, with ten chromothriptic-like events. CFA2, 5, 19, 20, 35, and 36 all showed an average of over four events (**b**). At least three of the cell lines displayed chromothriptic-like events on CFA2, 5, 7, 9, 10, 18, 20, 27 and X. Thus, complex chromothriptic-like activity is a common event in the UC cell lines, just as in primary tumors

Chromothriptic-like events were noted throughout the genome of all five of the cell lines, most notably on CFA 9 and 10 (>80 % of cell lines) (Fig. 2a). CFA 5, 10, 19, 35, and 36 had an average of over five chromothriptic-events per cell line, with CFA10 chromothriptic-like events occurring an average of 10 times per cell line (range = 1-20; Fig. 2b).

**Fig. 3:**
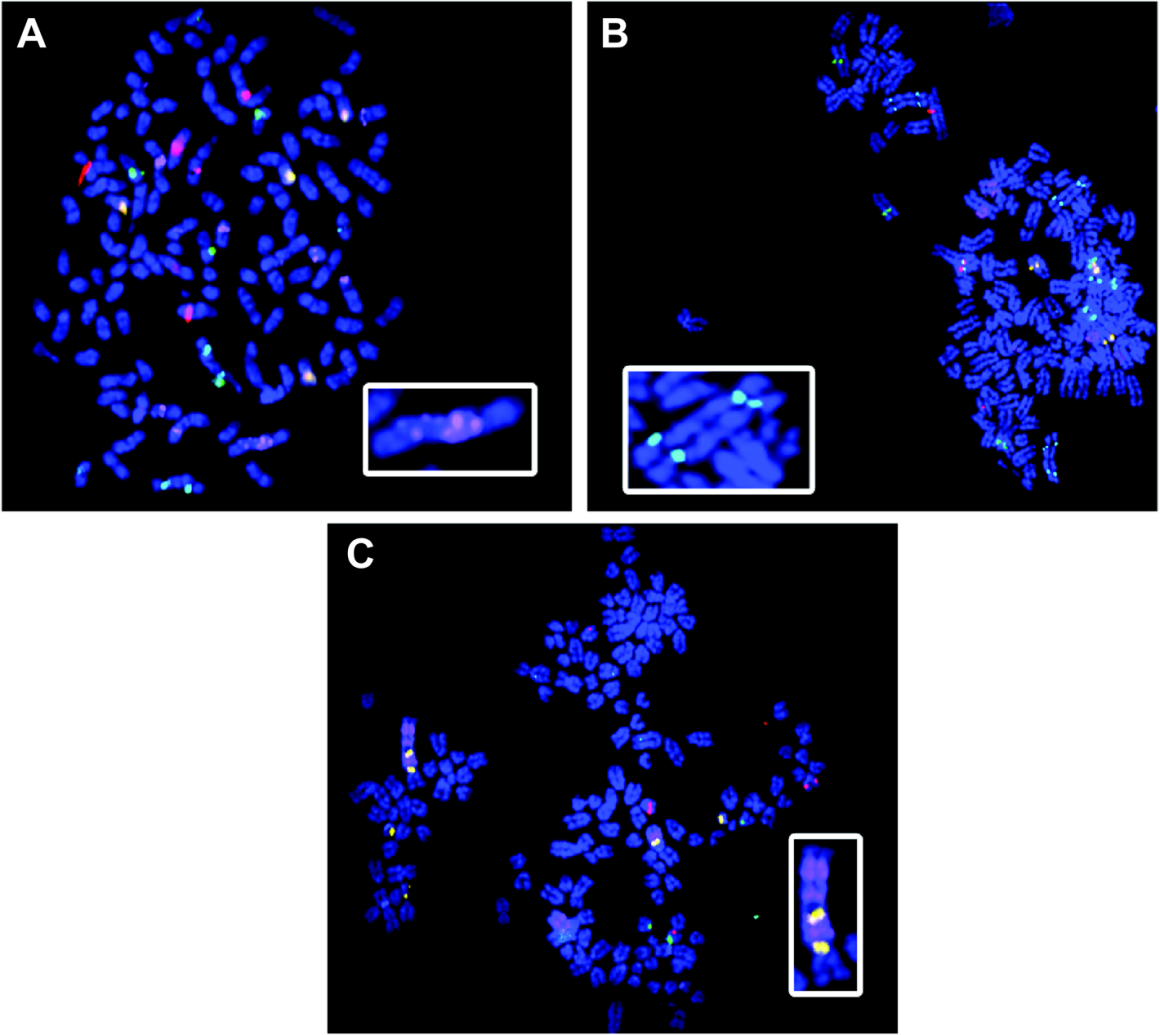
Structural Aberrations exist in oaCGH-flagged Regions. Tandem duplications, translocations, and fusions were noted throughout the karyotypes. According to oaCGH, CFA 10 (BAC probe CHORI-82:326H08; Cy5-labeled) shows a sharp spike of gain in the probed region (*MDM2*). FISH shows this spike might be attributed to tandem duplications of the locus along the chromosome (**a**). CFA 13 (BAC probe CHORI-82:BAC 186J06; Aqua-labeled) appears as two fused copies (**b**) multiple times within a karyotype, in addition to what appear to be normal copies, leading to an overall gain. Tandem duplication and locus translocation are both observed on chromosomes 36 (BAC probe CHORI-82:199F16, Cy5-labeled)/38 (BAC probe CHORI-82:328D10, Gold-labeled) (**c**). The pattern shows an overlap of duplication, in which a copy of the CFA 36 locus is flanked by CFA 38

Targeted FISH analysis of genomic loci identified as aberrant by oaCGH confirmed the presence of abnormal DNA copy number in individual cells and enabled enumeration of exact copy number (Table 5). No significant difference was observed among average oaCGH- and FISH-derived Log_2_ ratios (Table 5, *p* > 0.56 overall, Mann–Whitney *U* test), while correlation analysis showed a strong correlation between FISH- and oaCGH-determined Log_2_ ratios (*r* = 0.91). FISH enabled appreciation of hyperdiploid status in four of five cell lines (K9TCC-Pu-An, K9TCC-Pu-In, K9TCC-Pu-Mx, K9TCC-Pu-Sh), with average total chromosome number among 30 enumerated chromosome spreads ranging from 103 (K9TCC-Pu-In) to 134 (K9TCC-Pu-Mx) total chromosomes (Table 1). FISH further allowed identification and visualization of several recurrent structural aberrations involving interrogated regions (Fig. 3), including tandem duplications, translocations, chromosome fusions, and bi-armed chromosomes (Table 1). Tandem duplications were noted on CFA 10, 36, and/or 38 in all five cell lines (Fig. 3a & c), coincident with high level amplification indicated by the corresponding oaCGH profiles. An excess of 30 copies of the targeted locus of CFA 36 were noted in the K9TCC-PU-Mx line, confirming the expected Log_2_ > 4. The region probed on CFA 38 demonstrated a translocation to a position adjacent to the CFA 36 locus in two cell lines (K9TCC-PU-An and K9TCC-PU-Sh). In K9TCC-PU-Sh, tandem duplications of CFA 36, juxtaposed with tandem duplications of CFA 38, were present twice on a single aberrant chromosome structure (Fig. 3c). Additionally, centromeric fusion of CFA 13, resulting in a bi-armed aberrant chromosome, was noted in two lines (K9TCC-PU-An and K9TCC-PU-In; Fig. 3b). Other aberrantly metacentric chromosomes were noted in all five cell lines, affecting multiple chromosomes.

**Table 5:** Compiled FISH and oaCGH Log2 data

	CFA1	CFA6	CFA10	CFA12	CFA13	CFA19	CFA21	CFA33	CFA36	CFA38	CFA11	*p*
**K9TCC-Pu-An**												
oaCGH Log_2_	**−0.40**	**−0.50**	**2.94**	**−0.30**	**1.23**	**−0.96**	**−0.40**	**−0.60**	**0.60**	**1.20**	0.00	0.49
FISH Count	2.90	3.90	10.80	3.00	8.30	1.97	2.92	3.30	8.81	7.03	3.76	
FISH Log_2_	**−0.37**	0.05	**1.52**	**−0.33**	**1.14**	**−0.93**	**−0.36**	−0.19	**1.23**	**0.90**	0.00	
**K9TCC-Pu-In**												
oaCGH Log_2_	**−0.40**	**−0.53**	**1.40**	0.00	**0.52**	**−1.00**	**−0.40**	**−0.60**	**0.40**	**0.43**	0.00	0.32
FISH Count	3.40	3.73	11.00	4.07	5.62	2.50	4.10	3.00	6.87	6.77	3.79	
FISH Log_2_	−0.16	−0.02	**1.54**	0.10	**0.57**	**−0.60**	0.11	**−0.34**	**0.86**	**0.84**	0.00	
**K9TCC**												
oaCGH Log_2_	0.13	**−0.30**	**2.40**	**−1.00**	**0.96**	**−1.00**	0.00	0.07	0.00	**0.60**	0.00	0.65
FISH Count	2.23	2.10	6.96	1.13	3.96	1.06	2.00	2.60	1.97	2.03	1.97	
FISH Log_2_	0.18	0.09	**1.82**	**−0.80**	**1.01**	**−0.89**	0.02	**0.40**	0.00	0.04	0.00	
**K9TCC-Pu-Mx**												
oaCGH Log_2_	0.04	0.19	0.04	−0.20	**0.90**	0.00	−0.20	−0.22	**1.90**	**1.10**	0.00	0.76
FISH Count	3.80	4.80	4.30	2.88	6.61	3.90	2.50	3.57	11.97	5.71	3.88	
FISH Log_2_	−0.03	**0.31**	0.15	**−0.43**	**0.77**	0.01	**−0.63**	−0.12	**1.63**	**0.56**	0.00	
**K9TCC-Pu-Sh**												
oaCGH Log_2_	**−1.00**	**−1.30**	**1.30**	**−1.00**	**0.93**	0.00	**−1.80**	**−1.05**	0.20	**−1.30**	0.00	1.00
FISH Count	1.93	1.25	6.33	1.47	5.34	3.14	0.64	2.00	6.19	2.13	3.68	
FISH Log_2_	**−0.93**	**−1.56**	**0.78**	**−1.32**	**0.54**	−0.23	**−2.52**	**−0.88**	**0.75**	**−0.79**	0.00	

Log2 ratio values obtained from oaCGH analysis of cell lines and calculated by raw FISH data are shown. For each cell line, a Mann–Whitney *U* test was conducted to evaluate consistency of data between both analysis methods (*p* < 0.05). In all cases, the mean Log2 ratio obtained from oaCGH and FISH was not significantly different (*p* > 0.32 in all cases), validating oaCGH results and demonstrating the utility of oaCGH in large-scale copy number analysis. **Bolded** Log2 ratios demonstrate significant gains or losses (Log2 < −0.234 or Log2 > 0.2)

### Gene expression profiles of cell lines differ from those of normal urothelium

When profiled alongside healthy urothelium, the UC cell lines showed highly differential gene expression levels. Of 43,803 probes on the array, 8,688 probes, representing at least 3,303 Ensembl-annotated canine genes, displayed altered levels of expression with absolute fold change greater than 2.0 when normalized and compared to reference urothelium. Examples are shown in Table 4 and were selected based on the magnitude of fold change and potential relevance to tumorigenesis. Hierarchical expression clustering confirmed a vastly different expression landscape between tumors and control urothelium, each recognized as a separate outgroup based on DEGs (Additional file 2: Figure S1).

**Fig. 4:**
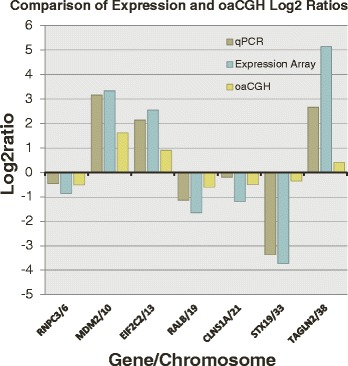
Gene expression arrays, qRT-PCR, and oaCGH results support one another and suggest a role of genomic copy number on mRNA levels. Seven differentially expressed genes located in copy number-aberrant regions (see Table 3) were selected for qRT-PCR analysis to validate microarray results. Correlation was very strong (*r* = 0.96) between the two methods and direction of fold change was in agreement for all genes, confirming the gene’s differential expression is true. In addition, comparison of qRT-PCR and oaCGH Log2 ratios revealed a strong correlation (*r* = 0.76), demonstrating the impact of genomic gene dosage on mRNA levels

qRT-PCR analysis of selected genes verified expression array results. All seven genes analyzed for each of the five cell lines had a direction of fold change in agreement with the expression data, with log fold change ratios calculated from qPCR results showing a very strong correlation to expression array log fold change (*r* = 0.96, Fig. 4). In addition, the magnitude of expression fold change calculated by qRT-PCR correlated strongly and positively with magnitude of copy number loss/gain, as calculated by FISH and oaCGH (*r* = 0.76, Fig. 4).

Using the DAVID tool for gene ontology (GO) and pathway analysis, DEG enrichment in functional pathways was evaluated. Among underexpressed genes (1859 total), the GO term “lipid metabolism” was the only GO term enriched (*p* = 0.03) in our dataset, with 189 genes underexpressed in the cell lines. Among overexpressed genes (1444 total), “DNA replication, recombination, and repair” was the most highly enriched term (*p* = 0.01), with 13 overexpressed genes. When evaluating all DEGs, “DNA replication and repair” was the single most commonly dysregulated functional ontology, represented by 10 % of all cell line DEGs.

KEGG pathway analysis in DAVID highlighted four pathways enriched for underexpressed genes: metabolism of xenobiotics by cytochrome p450, glutathione metabolism, drug metabolism, and fatty acid metabolism, resulting in a KEGG pathway clustering of these pathways with an enrichment score indicative of biological significance (Bonferroni = 0.05, enrichment score = 2.34). Additionally, 3.7 % of genes with five-fold or greater expression than normal urothelium (10 of 268) were involved in cell cycle regulatory pathways (*p* = 0.0004), as were 1.8 % (26 of 1444) of all upregulated genes (*p* = 0.00003). DAVID chromosome association analysis found 4.7 % of overexpressed genes (68 genes) were found on CFA 13 and 4.2 % on CFA 10 (61 genes). Similarly, 3.6 % (66 genes) of underexpressed genes were located on CFA 12 and 1.2 % on CFA 19 (23 genes).

## Discussion

Canine UC represents the most common genitourinary cancer in dogs and, due to the aggressive nature of the tumor, diagnosis conveys a guarded prognosis for the patient [1]. To date, curative therapies are lacking for UC, which is likely due in part to delayed tumor diagnosis and the lack of knowledge about the molecular basis of tumor pathogenesis. It is known, however, that canine UC closely resembles prognostically unfavorable invasive human UC, making research in canine bladder cancer potentially valuable in improving the outlook for human UC patients [6, 2, 1, 11]. As a result, a valid *in vitro* cell line model would be advantageous to both species and could potentiate investigation of more precise molecular mechanisms involved in UC pathogenesis, including the development of therapies to target them. Previously, five canine UC cell lines were established for *in vitro* study of the disease, and four of them retain tumorigenicity in mice [11]. With the potential to facilitate translational studies, care should be taken to determine that these cell lines provide a high clinical predictive value. Our study sought to evaluate: 1) the genomic similarity between primary UC and cell lines, 2) the genomic integrity of the cell lines over time, and 3) the gene expression landscape of canine UC cell lines.

Our results show that, although variation exists in the frequency of copy number aberrations between the cell lines and primary tumors, the most frequent copy number gains and losses are preserved in the cell lines, specifically loss of regions of CFA 19, and gain of regions of CFA 13 and 36 [4]. Among primary tumors, 100 % of the canine UC cases possess at least one of these aberrations, 94 % possess two, and 68 % possess all three aberrations, emphasizing the diagnostic potential of copy number aberrations and a potentially critical role of these chromosomes in tumorigenesis. Similarly, all five cell lines had at least one of these aberrations (100 %), four had at least two (80 %), and two had all three aberrations (40 %). When oaCGH-called aberrations among the 31 primary tumors and five cell lines were clustered, no cell line outgroup was seen, suggesting the cell lines were more like primary tumors than one another other and demonstrating a lack of culture artifact in their genomic profiles. Thus, the cell lines maintain the valuable aberrations of the primary tumor and provide an *in vitro* tool with which to further investigate the significance of these aberrations in canine UC.

The relevance of the gains on CFA13 and 36 are further emphasized by GISTIC analysis. GISTIC analysis, which focuses on regions containing genes repeatedly implicated in neoplastic transformation and tumor growth, highlighted only seven regions aberrant in the cell lines: amplified regions on CFA10, 36, and 38 and gains on CFA13. In primary tumors, however, GISTIC analysis implicated numerous regions, including the majority of those seen in the cell lines. These results not only emphasize the importance of genes highlighted by GISTIC analysis, but may also suggest that a number of the mutations seen in the primary tumor may be passenger mutations unessential for tumor maintenance in the cell lines. Thus, we may be able to utilize the less genomically complex cell lines to hone in on driver mutations crucial for UC pathogenesis and progression.

In addition to major chromosome aberrations, chromothriptic-like events represent another manifestation of copy number change. First described in 2011, chromothriptic-like events represent small genomic regions comprised of numerous adjacent and alternating copy number changes [19, 20]. Chromothriptic-like events were previously found to be prevalent throughout the genome of primary canine UC, particularly on CFA36, 10, 16, 4 and 7 [4]. Similarly, the cell lines show a high frequency and number of chromothriptic-like events throughout the genome, with 21 of 39 chromosomes demonstrating recurrent chromothriptic-like events in at least two cell lines. Among these are all of those observed at high frequency in primary tumors, except CFA16. The prevalence of chromothriptic-like events in the cell line genomes suggests inherent genome instability along with a massive chromosome shattering event. For example, tandem duplications observed on CFA10, 36, and 38, as well as translocations among these chromosomes (t(36;38)), may have arisen due to such chromothriptic events early in or leading to neoplastic transformation. Although arising from an unknown etiology, current hypotheses of chromothripsis in cancer implicate mutagenic exogenous compounds, which induce double-stranded DNA breaks throughout the genome, and chromosome missegregation [21, 22]. This type of chromosome shattering can lead to massive regional amplification, as well as losses and translocations, due to improper chromosome rejoining. Particularly when considering the potential etiology of risk-associated carcinogenic compounds, chromothripsis is a hugely interesting phenomenon in UC.

In addition to validating oaCGH-called aberrations, the ability to perform metaphase FISH on the cell lines allowed us to explore karyotypic reorganization occurring in canine UC, including recurrent tandem duplications responsible for CFA36 amplification and a CFA13 duplication and centromeric fusion often responsible for increases in CFA13. In our cell line analyses, copies of CFA10, 36, and 38 per cell were often too numerous to accurately count (>10-30 clearly distinguishable signals, particularly in K9TCC-Pu-Mx). Furthermore, in all cell lines, abnormally metacentric chromosomes were seen, suggesting prior mitotic dysfunction and/or breakage-fusion-bridge cycles and leading to the formation of new hybrid chromosomes.

The hyperdiploid karyotype of the cell lines, excluding K9TCC, further confirmed previous findings that canine UCs are often tetraploid (>50 % of UCs) [23]. Since oaCGH is blind to ploidy, the correlation between oaCGH status and copy number requires knowledge of ploidy status, making a copy number neutral control critical to appropriate copy number interpretation. CFA11 was identified as copy number neutral using oaCGH values and FISH analysis; CGH values suggestive of neither a loss nor gain (Log2 ≈ 0 for all cell lines) and chromosome structure appeared normal. In polyploid cell lines, four copies of CFA11 were consistently observed, supporting a tetraploid genome. Similarly, when oaCGH implied a copy number loss, one to three copies were observed, showing a relative, but often not absolute, loss of genetic material. In the case of CFA19, only in diploid K9TCC did the chromosome count consistently reflect an absolute copy number loss (n < 2). Relative to a duplicated genome, however, two copies of CFA 19 (K9TCC-PU-An) reflected a loss rather than a normal diploid number of Log_2_ = 0.

Cells were repeatedly passaged (>10 passages) in order to determine the stability of the UC genomic profile over time. Little change was evident in oaCGH profiles or chromosome structure of cell lines after passaging cells from early to mid-late stage. Only three regions on CFA1, 2, and 9 were significantly different between early and late passages, representing less than 0.006 % of the entire genome in length. Although the region on CFA9 contains no known genes, the regions on CFA1 and 2 contain genes potentially relevant to the culture environment: insulin-like growth factor 2 receptor (*IGF2R*, CFA1) and a protocadherin cluster (*PCDH*, CFA2). *IGF2R* and *PCDH* are both potential adaptations to the culture environment, allowing improved utilization of media sugars and improving culture flask adhesion, respectively. Nevertheless, such few aberrations acquired over 10+ passages represent a very minor effect of repeated passage and cell culture. This characteristic of the cell lines allows experimentation and analysis over time without concern of culture-induced changes, a valuable feature for translational studies.

We should note that several factors might influence our results. One is that cell lines are *ex vivo* models. Thus, some aberrations detected in cell lines may be due to the altered culture environment. We were unable to analyze the primary tumor from which the cell lines were derived, precluding our ability to detect initial culture-related genomic changes. Secondly, since oaCGH is a cell population-based analysis, it is plausible that rare aberrations evaded detection by oaCGH, but would become more prominent with continued neoplastic cell proliferation. However, our cell lines sustained the major copy number changes seen in primary tumors, even after ten or more passages, leading us to conclude cell lines represent a good model for UC *in vitro*.

Factors incriminated in the development of UC include exposure to cyclic amines, including carcinogens in cigarette smoke, estrogen, and cyclophosphamide, as well as obesity [7, 6, 2, 1, 24–27]. Interestingly, DAVID analysis of genes underexpressed in the UC cell lines revealed GO enrichment of genes essential to the metabolism of such lipophilic cyclic compounds. Genes underexpressed in the UC cell lines were also enriched in metabolic KEGG pathways, most notably pathways involved in xenobiotic metabolism. Decreased expression of metabolic genes suggests that toxic compounds are not being handled properly by the bladder epithelium, potentially leading to a buildup of corrosive compounds in the urine and increased exposure of the urothelium to carcinogens. In particular, carboxylesterase-1 (*CES-1*; CFA2; fold change = −633.5) is involved in metabolism of organophosphate and pyrethroid insecticides, both associated with UC development [28–30]. Similarly, decreased to undetectable levels of urothelial CES1 are associated with UC in humans [31]. In addition, CES enzymatic activity is highly variable among human individuals and may be associated with differences in canine breed susceptibilities [32, 33].

Of further interest, Cytochrome P450 2C19 (*CYP2C19*; CFA28; fold change = −8.1) is a potentially relevant metabolic gene central to the enriched cytochrome p450 KEGG pathway. CYP2C19 is one of the most important cytochrome p450 enzymes and is responsible for the metabolism of numerous cyclic amine xenobiotics, including the pyrethroid-derivatives of hydantoin present in common insecticides [34]. Similar to *CES1*, genetic polymorphisms of *CYP2C19* are associated with reduced metabolic capacity in humans, suggesting similar phenomena may occur among genetically distinct dog breeds [35, 36]. Our data corroborate previous speculations regarding the involvement of metabolic pathways in canine UC development and highlights a need for closer investigation.

Among genes overexpressed in the UC cell lines, GO terms were enriched for DNA replication, recombination, and repair. Similarly, KEGG analysis highlighted cell cycle regulator pathways, in which 4 % of genes with expression five-fold above that of normal urothelium were involved (1.8 % of all overexpressed genes). One of the most prominent overexpressed genes in our dataset and a key gene in DNA replication *and* cell cycle regulation--*PTTG1* (CFA4; fold change=+57.5). As a result of its functions as a sister chromatid securin, microtubule nucleation regulator, and AKT activator, among others, *PTTG1* overexpression potentiates tumor proliferation and invasion, as well as chromosome instability [37–39]. Interestingly, activating mutations in *FGFR3,* an upstream regulator of AKT activity, are implicated in constitutive activation of the AKT pathway commonly seen in human UC [40]. *PTTG1* has the potential to be a major driver of AKT activation in canine UC, providing a valuable therapeutic target and highlighting pathway dysregulation similarities in human and dog UC.

Our expression data also emphasized the potential impact of chromosome copy number on gene dosage and, therefore, gene expression. When analyzing genes located in regions of copy number aberration, it was shown that gene expression often varied directly with copy number change (*r* = 0.76). DAVID chromosome analysis showed enrichment of underexpressed genes on CFA19, which is frequently lost in UC cell lines. CFA 19 contains the gene for histamine N-methyltransferase (*HNMT*), a gene highly under expressed in our cell lines (fold change = −29.6). Although unreported in bladder cancer, reduced *HNMT* transcription has been reported in other human carcinomas and is posited to be involved in tumorigenesis [41]. Decreased levels of *HNMT* lead to local increases of angiogenesis-promoting *PTGS2 (COX2; CFA7). PTGS2* levels are increased in both canine and human UC, and this gene is similarly overexpressed in our cell line data (fold change = +76.9), providing a further mechanistic role for *COX-2* inhibitors in UC treatment [42, 43]. Additionally, *COX2* is a downstream effector of an activated AKT pathway, again suggesting a conservation of AKT overactivation in both canine and human UC development.

Similarly, gain of CFA 13 led to chromosome enrichment of overexpressed genes (4.7 % of overexpressed genes), including the oncogenic transcription factor *MYC*. Responsible for expediting the cell cycle and bypassing critical checkpoints, *MYC* has been implicated in numerous human cancers, including bladder cancer [44] and is overexpressed cell lines (fold change = +8.5), along with downstream targets cyclin D1 and D2 (fold change = +2.2 and +14.2, respectively). The increase in downstream targets suggests amplified *MYC* is not only over transcribed, but also translated and functionally active. Furthermore, our expression data suggests a high level of conservation in DEGs between human and canine UC (Table 6), providing a potentially valuable model of the human disease and reinforcing previous findings that canine and human UC are molecularly, clinically, and histopathologically similar.

**Table 6:** Gene expression microarray results

Gene	Location	Lines differentially expressing	Average fold change	Gene	Location	Lines with relative fold change ≥2 or ≤ −2	Average fold change
*CES1* ^*a*^	chr2	ALL	−633.5	*BIRC5* ^*a*^	chr9	In, An, Mx, K9TCC	+3.6
*HNMT*	chr19	In, Sh, Mx, K9TCC	−29.6	*RAF1* ^*a*^	chr20	ALL	+4.1
*DBC1* ^*a*^	chr11	ALL	−13.6	*CLU* ^*a*^	chr25	ALL	+5.5
*CDKN2B* ^*a*^	chr11	ALL	−11.8	*CCNG2* ^*a*^	chr32	ALL	+6.3
*FHIT* ^*a*^	chr20	ALL	−11.5	*MDM2* ^*a*^	chr10	In, Sh, An, K9TCC	+6.4
*CYP2C19* ^*a*^	chr28	ALL	−8.1	*MYC* ^*a*^	chr13	ALL	+8.5
*BCL2* ^*a*^	chr1	ALL	−6.6	*LAMB3* ^*a*^	chr7	ALL	+12.4
*SRPX* ^*a*^	chrX	In, An, Mx, K9TCC	−6.1	*TGFBR2* ^*a*^	chr23	ALL	+12.6
*FMO5* ^*a*^	chr17	ALL	−4.9	*CCND2* ^*a*^	chr27	ALL	+14.2
*SPARC* ^*a*^	chr4	ALL	−4.5	*AURKA* ^*a*^	chr24	ALL	+22.2
*PTEN* ^*a*^	chr26	ALL	−4	*TAGLN2* ^*a*^	chr38	ALL	+25.3
*BMP7* ^*a*^	chr24	An, Mx, K9TCC	−3.4	*PTTG1* ^*a*^	chr4	ALL	+57.5
*DAPK1* ^*a*^	chr1	An, Mx	−2.8	*CDK1* ^*a*^	chr4	ALL	+72.5
*VHL* ^*a*^	chr20	ALL	−2.1	*PTGS2* ^*a*^	chr7	ALL	+76.9

Numerous genes were dysregulated in the cell lines when compared to the expression profile of healthy canine urothelium. The genes listed were selected based on their magnitude of fold change, oncogenic potential, and relevance in human UC (see superscipt letter a). The specific cell lines differentially expressing each gene are listed (fold change ≥2 or ≤ −2 relative to the mean expression of two healthy urothelial samples)

Despite strong correlations between copy number gain/loss and differential gene expression (Fig. 4), it is not known if increased genomic dosage directly leads to increased transcript levels. In fact, *PABPC1*, a gene located in the region of shared high frequency gain on HSA 8/CFA 13 in human and canine UC, was actually underexpressed (average fold change = −2.6) in the cell lines [4]. However, *PABPC1* is highly expressed, at both mRNA and protein levels, in normal urothelium, potentially obscuring subtle differences in expression while reducing their physiologic significance [45, 46]. Additionally, while human expression studies have shown relative overexpression of *PABPC1* at the mRNA level, protein studies have shown a relative decrease in protein, espousing the need for protein evaluation alongside mRNA quantification [47]. We cannot discount the importance of epigenetic factors in determining expression. In addition, disruption of gene promoters and enhancers, as may occur due to tandem duplication, deletion, and/or translocation, may alter predicted gene expression. Post-transcriptional gene regulation may also play a role due to possible alterations in silencing RNA. Regardless, it is reasonable to assume genomic copy number plays a role in determining corresponding mRNA levels.

## Conclusions

The recurrence of specific genetic aberrations in UC emphasizes the importance of continued UC genomic research, particularly in predisposed dog breeds with reduced genetic variation and in association with prominent carcinogenic risk factors. A treatment found to be effective in canine cell lines, which then is effective in dogs, increases the clinical predictive value of the cell lines and, therefore, their value to biomedical research [11]. The preservation of primary tumor aberrations in the cell line model provides an accessible and accurate means of performing genetic experiments *in vitro*, making the cell lines a valuable resource for translational UC research. Future research should involve utilizing the UC cell lines in a functional manner to elucidate mechanisms of UC pathogenesis.

## Additional files

Additional file 1: Table S1. Comparisons analysis of primary tumors and cell lines. oaCGH data from primary tumors and cell lines were simultaneously analyzed and compared using the Comparisons algorithm (Nexus Copy Number, Biodiscovery). Few aberrant regions were evident in only the primary tumors or the cell lines without being present in the other. (XLSX 9 kb) Additional file 2: Figure S1. Clustered heat map of cell line differential expression*.* Unsupervised hierarchical clustering of gene expression profiles among healthy urothelium and cell lines highlighted numerous dysregulated genes among cell lines. Clustering along the Y-axis is by probe set, and clustering along the X-axis is according to expression profile similarity. Different colored blocks along the Y-axis group genes with a similar gene expression pattern. Above the X-axis, the top row of colored blocks denotes individual samples, while those below identify samples with similar gene expression profiles. Both healthy control samples were assigned the same color, demonstrating similar gene expression, while expression among the cell lines was more variable (different colored blocks). Large green blocks within the heat map represent down-regulated genes, while red are relatively up regulated when compared to the median expression among all samples. Healthy urothelium (columns 1 and 2) clearly segregates from the cell lines, suggesting altered gene expression among the cell lines. Heat maps were generated in Nexus Expression (Biodiscovery). (PPTX 242 kb)
