# Supplementary figures and images for: A cultured approach to canine urothelial carcinoma: molecular characterization of five cell lines
Source: Canine Genet Epidemiol. 2015 Sep 17;2:15. doi: 10.1186/s40575-015-0028-3 (PMC4579363; doi:10.1186/s40575-015-0028-3)

## Slide 1
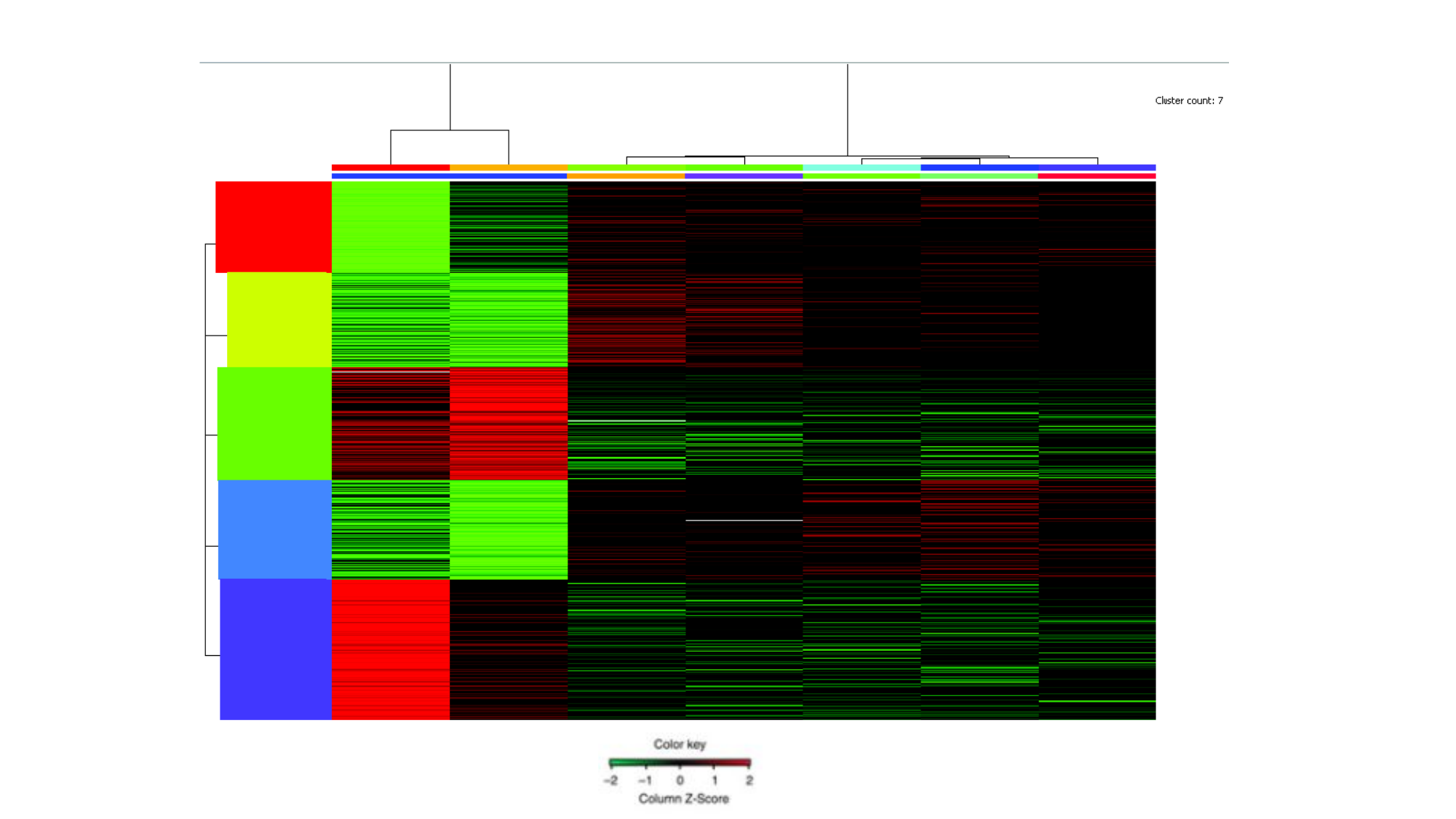

#

Supplement: Additional file 2: Figure S1. — Clustered heat map of cell line differential expression. Unsupervised hierarchical clustering of gene expression profiles among healthy urothelium and cell lines highlighted numerous dysregulated genes among cell lines. Clustering along the Y-axis is by probe set, and clustering along the X-axis is according to expression profile similarity. Different colored blocks along the Y-axis group genes with a similar gene expression pattern. Above the X-axis, the top row of colored blocks denotes individual samples, while those below identify samples with similar gene expression profiles. Both healthy control samples were assigned the same color, demonstrating similar gene expression, while expression among the cell lines was more variable (different colored blocks). Large green blocks within the heat map represent down-regulated genes, while red are relatively up regulated when compared to the median expression among all samples. Healthy urothelium (columns 1 and 2) clearly segregates from the cell lines, suggesting altered gene expression among the cell lines. Heat maps were generated in Nexus Expression (Biodiscovery). (PPTX 242 kb) [file 40575_2015_28_MOESM2_ESM.pptx]
